# The relationship between artificial intelligence and low-skilled employment in South Africa

**DOI:** 10.1016/j.heliyon.2024.e40640

**Published:** 2024-11-22

**Authors:** Fiyinfoluwa Giwa, Nicholas Ngepah

**Affiliations:** School of Economics, College of Business and Economics, University of Johannesburg, South Africa

**Keywords:** Low-skilled employment, South Africa, Vector error correction model, Artificial intelligence investment

## Abstract

As artificial intelligence (AI) continues to advance, its impact on employment is a topic of concern. In South Africa, where low-skilled labor forms a significant portion of the workforce, the integration of AI technologies raises questions about the future of employment opportunities and economic stability. This manuscript explores the relationship between AI adoption, low-skilled employment dynamics, and its implications using key economic indicators such as inflation, interest rates, and foreign direct investment (FDI). Employing the Vector Error Correction Model (VECM) approach from 2012Q1 to 2021Q4, the study's findings reveal a significant negative correlation between artificial intelligence and low-skilled employment in the long run. Granger causality tests reveal directional relationships, with AI investment unidirectionally causing low-skilled employment. As a policy implication, this study recommends implementing training programs to equip workers with the necessary skills to adapt to the evolving job market influenced by technological advancements. Additionally, it suggests monitoring the implementation of AI technologies and establishing policies to mitigate labor market disruptions.

## 1 Introduction

The integration of artificial intelligence (AI) into various industries has sparked discussions globally, particularly regarding its impact on employment. In the context of low-skilled workers in South Africa, the advent of AI presents both opportunities and challenges. As AI technologies advance, they offer potential solutions to streamline processes, improve efficiency, and enhance productivity. However, they also raise concerns about the displacement of human labor, particularly in sectors reliant on manual tasks [1].

The fourth industrial revolution, characterized by AI and automation, introduces unprecedented changes to the labor market. Tasks previously performed by low-skilled workers now face automation, leading to shifts in employment dynamics [2,3]. While some argue that AI will augment human capabilities and create new job opportunities, others fear significant job losses, especially among low-skilled workers. However, the extent to which AI adoption directly influences job opportunities and job quality among low-skilled workers in South Africa remains to be determined ([4]; [5]; [6]).

AI's impact on low-skilled employment in South Africa is of particular interest due to the country's socio-economic context. With high unemployment rates and a significant portion of the population possessing limited skills and education, the potential consequences of AI adoption are profound. The automation of routine tasks could exacerbate unemployment, deepen economic inequalities, and hinder inclusive growth [[7], [8], [9]].

This study aims to examine the relationship between AI and low-skilled employment in South Africa (main objective). It seeks to investigate the causal linkages between AI adoption and changes in low-skilled employment, exploring whether AI implementation directly influences job opportunities and job quality among low-skilled workers.

Through a comprehensive analysis of the intersection between AI and low-skilled employment in South Africa, this research aims to provide insights into the evolving labor market dynamics in the country. Additionally, it seeks to identify potential policy interventions to mitigate negative outcomes and promote inclusive economic development.

By shedding light on the challenges and opportunities presented by AI adoption, this study contributes to the limited existing literature on AI's socio-economic impacts especially for Africa. It provides valuable insights for policymakers, businesses, and stakeholders seeking to navigate the complexities of technology advancement while ensuring that the benefits are equitably distributed among all segments of society. The paper comprises six sections: introduction, literature review, theoretical review, methodology, results, and Discussion. The conclusion presents a summary of the study and policy implications.

## 2 Literature review

Artificial intelligence, a component of the Fourth Industrial Revolution (4IR), significantly impacts low-skilled employment in various ways. The effect of artificial intelligence on job opportunities for low-skilled workers is multifaceted, with two potential scenarios. AI often complements human labor, potentially increasing demand for low-skilled workers and creating job opportunities. However, on the other hand, the superior efficiency of AI-enabled robots in performing tasks could lead to job displacement, as they can complete tasks rapidly and cost-effectively. According to Badiuzzaman and Rafiquzzaman [10], certain types of low-skilled jobs are more susceptible to AI technology. These typically involve repetitive physical tasks, particularly in industries such as manufacturing and retail trade, where lower and middle-skill professions are also at higher risk of being automated. Thus, the integration of artificial intelligence and automation in the workforce presents both opportunities and challenges for low-skilled workers, shaping the future landscape of employment.

The impact of artificial intelligence (AI) and automation on employment, particularly for low-skilled workers, is a topic of extensive study and debate. Zierahn et al. [11] found that approximately 9 % of jobs across 21 OECD countries could be automated, with lower-skilled workers bearing the brunt of potential job losses. Similarly, Ozcan (2019) noted an increase in demand for skilled labor alongside a decline in opportunities for low-skilled workers due to automation. Smith and Anderson [12] underscored the widespread belief among technology experts that robots could increasingly perform standardized labor tasks, posing a threat to various job sectors.

Graetz and Michaels [13] analyzed data from the EU KLEMS database and found a negative correlation between robotization and low-skill employment across multiple industries and countries. Zhang et al. [14] corroborated these findings in China, showing that high-skilled labor benefited more from industrial robot adoption. Balsmeier and Woerter [15] also observed in Switzerland that the adoption of digital technology led to increased employment of high-skilled workers while reducing opportunities for low-skilled workers.

Conversely, Ma et al. (2022) discovered a nuanced impact of AI development in China, where innovation decreased low-skilled employment while creating opportunities for high-skilled workers. Tang et al. [16] found a significant rise in highly skilled workers in firms adopting robots, although the adoption had minimal effects on low-skilled workers. Similarly, Klenert et al. [17] found no evidence that robots decrease the proportion of low-skilled workers in Europe.

The studies reviewed indicate a complex relationship between artificial intelligence (AI), automation, and low-skilled employment. While there is consensus that automation poses a threat to certain low-skilled jobs, the impact varies across industries and regions. Some findings suggest that AI adoption leads to declining opportunities for low-skilled workers, with high-skilled labor benefiting more from technological advancements. However, other studies reveal a nuanced effect, where innovation decreases low-skilled employment while creating new job opportunities for high-skilled workers. Overall, the results emphasize the need for further research to understand the full implications of AI on low-skilled employment and to develop targeted policies to mitigate potential job displacement.

## 3 Conceptual framework

The Routine-Biased Technological Change (RBTC) theory by Autor, Levy, and Murnane [18] is a framework that seeks to explain the impact of technological advancements. RBTC theory posits that technological change affects different types of tasks and jobs to varying degrees, leading to changes in employment and wage inequality. The theory highlights the role of routine tasks in shaping labor market dynamics. RBTC theory argues that technological change, particularly automation, exhibits a bias towards routine tasks, both cognitive and manual. Automation can decrease the demand for workers performing routine tasks, often associated with low-skill levels.

RBTC theory suggests that technological change is not uniform across all tasks and occupations but has a biased effect on routine tasks. Routine tasks follow explicit rules and can be easily automated or outsourced to computers and machines. Examples of routine tasks include data entry, assembly line work, and some administrative jobs. According to RBTC theory, technological progress tends to complement non-routine tasks involving problem-solving, creativity, and complex decision-making. Non-routine tasks are typically associated with higher-skilled and higher-paying occupations, such as professional, managerial, and creative roles [19].

In contrast, routine tasks are susceptible to automation and technological substitution. As technology advances, routine tasks become more easily automated, leading to a decline in demand for workers performing these tasks. This automation process can lead to job displacement and unemployment for workers engaged in routine occupations [20].

RBTC theory argues that the displacement of routine tasks can result in labor market polarization. On one end, high-skilled workers in non-routine occupations experience an increase in demand and wages due to their complementary relationship with advancing technology. Conversely, low-skilled workers engaged in routine tasks face job losses and wage stagnation or decline as machines substitute their work. This polarization can contribute to income inequality as the wages of high-skilled workers rise faster than those of low-skilled workers. The theory suggests that middle-skill jobs that involve routine tasks, such as manufacturing and administrative work, are most vulnerable to technological displacement (Jung and Mercenier, 2014).

The RBTC hypothesis posits that the production process is characterized by the definition of tasks. Job tasks are assigned to workers or capital based on three factors: the level of automation, the degree of separability from other tasks, and the cost comparison between machines and humans. In this context, the term 'machines' encompasses various forms of technology, including hardware, software, and their amalgamations, such as robots. A crucial aspect of this framework is the differentiation between tasks and skills. Acemoglu and Autor [21] define a task as a unit of work that generates output in the form of goods and services. They also define a skill as the capabilities possessed by a worker to perform different tasks. Tasks performed by workers in their jobs can be subject to change due to technological advancements and fluctuations in the cost of labor compared to capital.

In Routine-Biased Technological Change (RBTC) theory, tasks are categorized based on routine and non-routine nature. RBTC theory suggests that technological change affects different types of tasks to varying degrees, leading to changes in the demand for different skill levels and job polarization. These categories include manual-routine, manual-non-routine, cognitive-routine, cognitive-non-routine interactive, and cognitive-non-routine analytical tasks. These task categories provide a framework for understanding how technological change affects the demand for different tasks and the corresponding impact on employment and skills. RBTC theory suggests that routine tasks, both manual and cognitive, are more susceptible to automation, while non-routine tasks, particularly those involving complex cognitive skills and social interactions, are less easily automated and more complementary to technology [22].

The equation for the production function can be modified within the RBTC theory to incorporate the impact of technological progress on the labor market. Let us examine a simplified production function that establishes a relationship between output (Y) and the factors of production, namely labor (L) and capital (K):

1 Y=F(L,K)

The equation presented herein illustrates the production function, denoted by the symbol F, which demonstrates how labor and capital inputs are combined to generate output. Additionally, it is worth considering a basic production function that establishes a relationship between output (Y), labor (L), and technology (A): This can be written as:

2 Y=F(L,A)

The RBTC theory posits that technological advancements might have a disproportionate impact on routine jobs, altering the demand for labor and its composition within the labor market.

In order to account for this bias, the production function equation may be expanded to encompass the influence of routine-biased technical progress. A frequently employed strategy is the inclusion of an additional variable termed "task content" (T), which denotes the degree of regularity or irregularity associated with the labor activities being executed:

3 Y=F(L,A,T)

The parameter T is utilized to quantify the impact of technology advancements on the nature and characteristics of tasks. The phenomenon under consideration has a discernible effect on the efficiency of labor and exhibits variability across various work categories. The demand for routine jobs is prone to drop due to automation or substitution, influencing their prevalence. Conversely, non-routine work may acquire greater value or complement technology, resulting in a rise in demand.

Furthermore, the production function equation can be reformulated to establish a direct relationship with the labor market, making labor the focal point of the formula. In order to integrate the RBTC theory and its ramifications for the labor market, it is possible to reframe the production function, as denoted by equation (1), in relation to labor productivity (P) instead of output (Y). Hence, the first equation can be represented as:

4 P=Y/L

Subsequently, labor productivity can be represented as a mathematical function incorporating routine-biased technological change (T), labor (L), and technology (A). Which is expressed as:

5 P=F(T,L,A)

In this modified equation, P denotes labor productivity, T represents the impact of routine-biased technological change on labor productivity, L denotes labor, and A represents technology. All this captures how technological advancements affect the relative demand for routine and non-routine tasks denoted by the parameter P.

## 4 Methodology

In an attempt to investigate the subject matter of this paper, this study adopts and modifies the study of Zhao (2022), who analyzed technology innovation effect on employment in China's tertiary sector. The regression analysis of this paper takes the linear form:

6 $$LSEMP=\alpha_{0}+\beta_{1}{AI}_{t}+\beta_{2}\;{\mathrm{INF}}_{t}+\beta_{3}{\mathrm{INT}}_{t}+\beta_{4}{FDI}_{t}+\varepsilon_{t}$$

LoSEMP, AI, and INF denote Low-skilled employment, artificial intelligence investment, and inflation, respectively. The inclusion of inflation, foreign direct investment, and interest rates in the analysis is crucial because these factors have been shown to influence low-skilled employment. Zhao [23] highlights the significance of foreign direct investment in shaping individuals' employability. Moreover, Hami and Orhan (2023) underscore how interest rates impact borrowing costs for companies and firms, subsequently affecting their operations and potentially influencing their hiring decisions. Thus, considering these economic variables provides a comprehensive understanding of the factors contributing to low-skilled employment rate fluctuations.

In logarithm, equation (6) can be expressed as follows:

7 $$InLoSEMP=\alpha_{0}+\beta_{1}In{AI}_{t}+\beta_{2}\;{\mathrm{INF}}_{t}+\beta_{3}{\mathrm{INT}}_{t}+\beta_{4}{INFDI}_{t}+\varepsilon_{t}$$

Where InLoSEMP represent logarithm for low-skilled employment, $In{AI}_{t}$ represent logarithm for artificial intelligence, and ${INFDI}_{t}$ denotes logarithm for foreign direct investment. On the other hand, inflation (INF) and interest rates $\left({\mathrm{INT}}_{t}\right)$ are not in logarithm because these variables are already in percentages and some of the figures are negative, hence the logarithm of then variables may not be possible.

According to the RBTC economic theory, AI is predicted to have a negative effect on low-skilled employment. The same holds for inflation and interest rate, according to Hami and Orhan (2023) and Adeyemi [24]. On the other hand, foreign direct investment will positively impact low-skilled employment [23].

### 4.1 Variables and data Source

This study utilizes data from the OECD and Quantec databases for the period spanning from the first quarter of 2012 to the fourth quarter of 2021. The study utilizes quarterly data. Table 1 presents an overview of the dataset, including the measurements taken and the definitions of the variables used in the study.

**Table 1:** Dataset and measurement.

**Variables**	**Definition of variables**	**Description**	**Expected Signs**	**Source**
Low-Skilled Employment (LoSEMP)	Jobs or professions classified as low-skilled need little formal education, specialized training, or specialist technical abilities. These positions typically entail simple, repetitive duties that can be acquired relatively quickly and do not require a high level of education or professional experience.	Low-Skilled employment formal sector (millions)	Positive	Quantec database
AI investment (AI)	AI is computer science focused on creating machines with human-like intelligence. It involves designing algorithms that can analyze data, learn, make decisions, solve problems, and simulate human cognitive abilities.	USD millions	Negative	OECD
Inflation (INF)	A steady rise in the average price of goods and services over an extended period of time is referred to as inflation. As more money is needed to purchase the same amount of goods and services, it indicates a decline in the buying power of a unit of currency.	Annual percentage	Negative	Quantec database
Interest Rates (INT)	The interest rate is the rate at which the central bank lends money to commercial banks. It affects financial transactions like loans, mortgages, credit cards, savings accounts, and government bonds.	Lending Rate: All domestic private sectors (percentage)	Negative	Quantec database
Foreign direct investment (FDI)	FDI is the act of an individual, organization, or entity from one country investing in another country to establish a lasting interest or significant influence in the management and operations of a business enterprise in the host country.	Foreign assets: Total direct investment (million rands)	Negative	Quantec database

### 4.2 Estimation technique

The Vector Error Correction Model (VECM) is an extension of the Vector Autoregressive (VAR) model that incorporates the concept of cointegration to mitigate the spurious regression problem that may arise when dealing with non-stationary time series. The Vector Error Correction Model (VECM) includes an error-correction term, which captures how variables adjust toward their long-term equilibrium following temporary deviations. The error-correction term is a corrective mechanism that compels the variables to revert to their cointegrating relationship. The error-correction term is derived from the cointegrating vectors acquired through the Johansen test. The Vector Error Correction Model (VECM) incorporates the utilization of the first differences of the variables, lagged differences of the variables, and the error-correction term as constituents of the model. Vector Error-Correction Models (VECM) necessitate the utilization of first-difference I (1) variables instead of the initial non-stationary variables. The general form of a VECM can be specified as follows:

8 $${\Delta y}_{t}=v+{\Pi Y}_{t-1}+\theta_{1}{\Delta y}_{t-1}+\theta_{2}{\Delta y}_{t-2}+\ldots +\theta_{p-1}{\Delta y}_{t-\left(p-1\right)}+\mu_{t}$$

Equation (7) can be expressed using the VECM model as:

9 $${\Delta InLSEMP}_{t}=v+{\Pi Y}_{t-1}+\theta_{1}{\Delta InAI}_{t-1}+\theta_{2}{\Delta \mathrm{INF}}_{t-2}+\theta_{3}{\Delta \mathrm{INT}}_{t-3}+\theta_{4}{\Delta InFDI}_{t-4}+\theta_{p-1}{\Delta y}_{t-\left(p-1\right)}+\mu_{t}$$

## 5 Empirical result and Discussion

In this section, the result of the study is discussed, beginning with the descriptive test, unit root test, optimum lag, Johansen cointegration test, Vector Error Correction (VEC) model, Granger causality, impulse response function, variance decomposition, and the diagnostic tests. The research questions include.

• Does artificial intelligence have a relationship with low-skilled employment in South Africa?
• Is there a causal relationship between artificial intelligence and low-skilled employment in South Africa?
• Do other economic factors - inflation, interest rate, and foreign direct investment affect low-skilled employment in South Africa?

The main objective of the study is.

• To analyze the long and short-run relationship between artificial intelligence and low-skilled employment in South Africa

The specific objectives of the study are as follows.

• ∗To examine the causal association behavior between artificial intelligence and low-skilled employment in South Africa
• ∗To investigate the impact of inflation, interest rate, and foreign direct investment on low-skilled employment in South Africa

### 5.1 Descriptive statistics

The descriptive statistics for the complete sample of the study are presented in Table 2. The study used quarterly data from 2012 to 2021. Artificial intelligence exhibits a mean of 15.999, a moderate left-skewness of −1.182, and a relatively high kurtosis of 3.668. The interest rate exhibits a high kurtosis of 7.284, indicating a relatively peaked distribution. It is also characterized by a negative skewness of −2.408, indicating a longer left tail. Additionally, the interest rate has a high mean value of 42.513. Low-skilled employment and foreign direct investment have similar means (14.967 and 14.708, respectively) and exhibit moderate left-skewness (−0.496 and −0.860, respectively). They also display low to moderate kurtosis (1.884 and 2.475, respectively). Inflation exhibits a kurtosis of 2.353, indicating a relatively low level of peakedness in its distribution. It also demonstrates moderate left-skewness, with a value of −0.359. Additionally, inflation has a relatively low mean of 5.157.

**Table 2:** Descriptive statistics.

**Variable**	**Mean**	**Median**	**Maximum**	**Minimum**	**Std. Dev**	**Skewness**	**Kurtosis**	**J-B Stat**	**Prob**	**Ob**
**LoSEMP**	14.967	14.977	15.021	14.894	0.039	−0.496	1.884	3.441	0.178	40
**AI**	15.999	16.727	18.871	12.345	1.519	−1.182	3.668	10.069	0.006	40
**INF**	5.157	5.150	6.700	3.200	0.935	−0.359	2.353	1.556	0.459	40
**INT**	42.513	47.540	51.710	0.000	14.758	−2.408	7.284	69.256	0.000	40
**FDI**	14.708	14.874	15.119	13.764	0.407	−0.860	2.475	4.987	0.082	40

The five variables have a range of values that extends from a minimum of 0.000 to a maximum of 51.710. All variables' distributions are skewed negatively because all their skewness values are negative. In addition, the Jarque-Bera test demonstrates that the dataset follows a normal distribution, which indicates that it is suitable for empirical investigation.

### 5.2 Unit root test

Vector Error-Correction Models (VECM) require the utilization of first-difference I (1) variables. Differencing is primarily employed to eliminate the trend component from data and achieve stationarity. Stationarity is essential in time series analysis as it simplifies the modelling process and maintains the constancy of statistical properties over time. Hence, the VECM model can be efficiently applied with first-difference variables. The Dickey-Fuller GLS and Phillips-Perron tests show that all the series under examination are integrated of order one (at first difference). Since none of the variables show I (0) or I (2) characteristics, the stationarity test results strongly support using the VECM model in this study.

Table 3 demonstrates the results of the unit root test, revealing that low-skilled employment (LoSEMP), artificial intelligence (InAI), inflation (INF), interest rate (INT), and foreign direct investment (InFDI) demonstrate stationarity at the first difference (I(1)). As such, we fail to reject the null hypothesis, indicating that these variables are stationary at the first difference level. Following this analysis, the lag length criteria are examined to determine the appropriate lag length for the model, which is crucial for the Vector Error Correction Model (VECM) estimation.

**Table 3:** Stationarity tests.

**Dickey-Fuller GLS**					**Phillips-Perron**				
**Variables**	Stationarity of Variables at level		Stationarity of Variables at first difference		Stationarity of Variables at level		Stationarity of Variables at first difference		
	No Trend	Trend	No Trend	Trend	No Trend	Trend	No Trend	Trend	
**LLoSEMP**	0.1035	0.0728∗	0.0369∗∗	0.0156∗∗	0.3432	0.8427	0.0379∗∗	0.0178∗∗	I(1)
**LAI**	0.0461	0.1912	0.0000∗∗∗	0.0000∗∗∗	0.0547	0.2242	0.0000∗∗∗	0.0000∗∗∗	I(1)
**INF**	0.1434	0.0123∗∗∗	0.0000∗∗∗	0.0000∗∗∗	0.3528	0.2371	0.0000∗∗∗	0.0000∗∗∗	I(1)
**INT**	0.2016	0.1119	0.0000∗∗∗	0.0000∗∗∗	0.0006∗∗∗	0.0087∗∗∗	0.0000∗∗∗	0.0000∗∗∗	I(1)
**LFDI**	0.6304	0.1804	0.0599∗	0.0054∗∗∗	0.4196	0.8429	0.0246∗∗	0.0065∗∗∗	I(1)

Note: Significance levels are 1 %, 5 %, and 10 %, denoted by ∗∗∗, ∗∗, and ∗, respectively.

### 5.3 Lag length criteria

Table 4 illustrates the lag-order selection of the model, showcasing various criteria such as final prediction error (FPE), Akaike's information criterion (AIC), Schwarz's Bayesian information criterion (BIC), and the Hannan and Quinn information criterion (HQIC). Among these criteria, the lag length marked with an asterisk is considered preferable as it represents the optimal choice for the model.

**Table 4:** Lag length criteria.

**Lag**	**LogL**	**LR**	**FPE**	**AIC**	**SC**	**HQ**
**1**	17.4625	NA	1.1006∗	0.4568∗	1.5905∗	0.8382∗
**2**	34.6465	23.9534	1.9206	0.9305	3.1979	1.6934
**3**	45.1056	11.4099	6.0306	1.8117	5.2129	2.9561

Note: Significance levels are 1 %, 5 %, and 10 %, denoted by ∗∗∗, ∗∗, and ∗, respectively.

The findings presented in Table 4 indicate that all four criteria (FPE, AIC, SC, and HQ) favor the selection of lag order one. This consensus across the criteria suggests that lag one is the most favorable choice according to each criterion. Incorporating additional lags into the model might result in overfitting and not substantially enhance forecasting accuracy or model simplicity. Additionally, the Johansen cointegration result is presented in Table 5.

**Table 5:** Johansen cointegration test.

**Unrestricted Cointegration Rank Test (Trace)**				
Hypothesized No. of CE(s)	Eigenvalue	Trace Stat	0.05 Critical Value	Prob. Critical Value
None ∗	0.740338	108.5037	69.81889	0.0000
At most 1 ∗	0.537766	62.65894	47.85613	0.0011
At most 2 ∗	0.463972	36.42165	29.79707	0.0075
At most 3	0.277061	15.22032	15.49471	0.0550
At most 4 ∗	0.115936	4.189694	3.841465	0.0407
**Unrestricted Cointegration Rank Test (Max-eigenvalue)**				
Hypothesized No. of CE(s)	Eigenvalue	Trace Stat	0.05 Critical Value	Prob. Critical Value
None ∗	0.740338	45.84473	33.87687	0.0012
At most 1	0.537766	26.23728	27.58434	0.0736
At most 2 ∗	0.463972	21.20133	21.13162	0.0489
At most 3	0.277061	11.03063	14.26460	0.1527
At most 4 ∗	0.115936	4.189694	3.841465	0.0407

### 5.4 Johansen cointegration test

The Johansen cointegration test ascertains the existence of cointegration within a collection of time series variables. The procedure entails estimating models with varying cointegrating relationships and comparing likelihood ratio test statistics to ascertain suitable cointegrating vectors. The Johansen cointegration result is presented in Table 5.

The Johansen Cointegration test results utilize the Trace and Max-eigenvalue tests to determine the number of cointegrating relationships within the data. Both tests indicate evidence of cointegration among the examined variables. Specifically, the Trace test shows a high level of statistical significance, while the Max-eigenvalue test results are also significant but at a somewhat lower level. These findings collectively suggest the presence of long-term relationships among the time series variables.

### 5.5 Vector error correction model (VECM) estimation result

VECM represents an expansion of the Vector Autoregression (VAR) model. It is specifically designed to effectively examine the intricate dynamics of variables that exhibit transient fluctuations in the short-term and enduring relationships in the long term. The VECM estimated result is presented in Table 6.

**Table 6:** VECM estimation result.

VECM Short-Run Result			
Variables	**Coefficient**	**Standard Error**	**T.Stat**
**D((LLoSEMP-1))**	1.000000		
**D (LAI (-1))**	−0.110244	0.01317	−8.37173
**D (INF (-1))**	−0.185778	0.03524	−5.27188
**D (INT (-1))**	0.005873	0.00244	2.41096
**D (LFDI (-1))**	0.012907	0.04360	2.29606
**C**	0.020092		

VECM Long-Run Result			
**Variables**	Coefficient	Standard Error	T.Stat

**ECT**	−0.010617	0.00462	−2.29625
**D (LAI (-1))**	−0.089286	0.08160	−2.24004
**D (INF (-1))**	−0.120005	0.00991	2.01887
**D (INT (-1))**	−0.046010	0.02196	0.07632
**D (LFDI (-1))**	0.035148	0.02073	−2.69577

The long-run model can be described as follows, with low-skilled employment (LoSEMP) as the target variable:

$${InLoSEMP}_{t}=-{0.089InAI}_{t}-0.120\;{\mathrm{INF}}_{t}-{0.046\mathrm{INT}}_{t}+{0.035InFDI}_{t}{-0.011ECT}_{t}$$

The coefficient of −0.110244 represents the short-run effect of artificial intelligence on low-skilled employment. The negative sign indicates an inverse relationship, meaning that an increase in artificial intelligence investment is associated with a decrease in low-skilled employment in the short run in South Africa. Likewise, the long-run result reveals a negative relationship between artificial intelligence investment and low-skilled employment. A 1 % increase in AI will decrease low-skilled employment (LoSEMP) by 8.9 %. In other words, it is anticipated that as the use of artificial intelligence technology grows over time, low-skilled jobs will decline. Also, both the t-statistics of the long and short run estimate are above the critical value of two; hence they are both significant. The negative coefficient, in the long run, further indicates that the adoption of artificial intelligence can result in the automation of tasks that workers with lower skill levels typically carry out. This phenomenon can lead to significant shifts in the labor market, potentially resulting in decreased demand for workers with lower skill levels in specific industries or sectors [25,26]. According to Oosthuizen [27], Given the increasing acceptance of artificial intelligence, an increased need for competent employees who possess the requisite technical knowledge to create, oversee, and execute AI systems may arise. This phenomenon may result in a discrepancy between the skills possessed by workers and the skills demanded by employers, resulting in higher unemployment rates among low-skilled individuals.

Also, the decline in demand for low-skilled labor and the simultaneous rise in demand for high-skilled labor may alter wage dynamics. Low-skilled workers may experience stagnant or declining wages due to decreased demand, whereas high-skilled workers possessing expertise in AI-related domains may observe an increase in their wages [28]. Furthermore, the impact of AI adoption is expected to vary across industries and sectors in South Africa. Certain industries may witness substantial increases in productivity, while others may encounter difficulties in adjusting, potentially resulting in changes in employment trends and disproportionately impacting workers with lower skill levels [29].

The negative relationship between artificial intelligence investment (AI) and low-skilled employment (LoSEMP) is consistent with the findings of Ma et al. [30], who examined the progression of AI development and employment skill structure in China between 2003 and 2017. Findings revealed a decrease in low-skilled labor employment due to the compensation effect. Also, the result aligns with the Routine-Biased Technological Change (RBTC) theory which suggests that automation can decrease the demand for workers performing routine tasks, which are often associated with low-skill levels.

The long-run and short-run inflation outcomes suggest a significant inverse correlation between inflation and low-skilled employment. Higher inflation is negatively correlated with low-skilled employment, both in the short and long run. A 1 % increase in AI will decrease low-skilled employment (LoSEMP) by 12 % in the long run and 18.6 % in the short run, respectively. This result is consistent with the findings of Salazar [31] study demonstrated that higher inflation has an adverse impact on employment creation, whether skilled or unskilled.

Inflation has different economic implications for South Africa. High inflation rates can cause uncertainty among businesses and investors, potentially resulting in decreased investment and limited expansion initiatives. Insufficient investment can hinder the expansion of businesses, thereby impeding the creation of employment opportunities, particularly for individuals with low skills. Inflation can influence the labor market by impacting job availability and quality for low-skilled workers. High inflation rates can cause economic uncertainty and diminish business confidence, which may result in postponed hiring decisions and hinder job creation [32].

Furthermore, inflation can have varying effects on different sectors of the South African economy. Industries that heavily depend on low-skilled workers, such as agriculture and specific manufacturing sectors, may encounter difficulties managing inflationary pressures. These sectors may face decreased hiring or job losses as businesses adapt to the evolving economic circumstances [33].

The result of interest rate reveals a positive and significant relationship with low-skilled employment in the short run. The long-run result is, however, insignificant. Every 1 % increase in interest rate increases low-skilled employment by 0.5 % in the short run. This implies that higher interest rates have the potential to impact economic activity negatively and result in a decline in low-skilled employment. Increased interest rates have the potential to influence borrowing expenses, business investments, and consumer expenditure, thereby impacting job creation across different sectors, including those that employ individuals with limited skills [34]. This result aligns with the findings of Oesch [35], who found that low real interest rates are associated with significantly less low-skilled unemployment in the OECD countries.

The result of foreign direct investment in Table 6 reveals a positive and significant relationship with low-skilled employment in the short and long run. Every 1 % rise in FDI will lead to a 1.3 % and 3.5 % increase in low-skilled employment in the short and long run, respectively. This implies that foreign direct investment has the potential to significantly contribute to the creation of employment opportunities for individuals with low skills. Foreign investors frequently create employment opportunities in diverse sectors, including those that employ low-skilled individuals, when establishing or expanding their operations in the host country. FDI inflows are frequently linked to enhanced economic growth and increased productivity. Economic growth typically leads to increased labor demand, encompassing low-skilled workers, contributing to favorable employment rates [36]. This finding is consistent with Le et al. [37], who examined foreign direct investment and employment in Vietnam. Their result reveals that FDI contributes to higher employment in Vietnam.

The negative coefficient of the error correction term (ECT) suggests that low-skilled employment tends to return to its long-run equilibrium level following short-term shocks or deviations. The error correction term is responsible for restoring equilibrium in the long run between low-skilled employment and other variables within the system. The coefficient (−0.010617) represents the rate at which low-skilled employment moves towards its long-term equilibrium level. A coefficient with a larger absolute value implies a more rapid adjustment process, while a smaller absolute value indicates a slower adjustment speed. Also, a statistically significant error correction term indicates a stable long-term relationship among the variables in the VECM system.

### 5.6 Granger causality test

Granger causality examines the ability of past values of one time series variable to offer information or predict future values of another variable. The Granger causality result is presented in Table 7.

**Table 7:** Granger causality test.

**Null hypothesis**	**Chi-Square**	**P-value**	**Nature of Direction**
**AI**→**LoSEMP**	7.658	0.0057∗∗∗	AI → LoSEMP
**LoSEMP**→**AI**	0.940	0.3320	None
**INF**→**LoSEMP**	13.041	0.0003∗∗∗	INF → LoSEMP
**LoSEMP**→**INF**	2.260	0.1327	None
**INT**→**LoSEMP**	0.549	0.4585	None
**LoSEMP**→**INT**	0.0373	0.8468	None
**FDI**→**LoSEMP**	4.7374	0.0295∗∗	FDI → LoSEMP
**LoSEMP**→**FDI**	4.5573	0.0328∗∗	LoSEMP → FDI

Note: Significance levels are 1 %, 5 %, and 10 %, denoted by ∗∗∗, ∗∗, and ∗, respectively.

Table 7 discusses the Granger causality of the model. The result reveals that the directional relationship between artificial intelligent investment and low-skilled employment is uni-directional. The result suggests that past variations or oscillations in the artificial intelligence variable possess valuable insights for forecasting forthcoming changes in low-skilled labor. Hence the advancements in artificial intelligence (AI) could influence the extent of work opportunities available for individuals with lower skills. Additionally, the progress or modifications in artificial intelligence technology may impact the demand for low-skilled labor. The increasing adoption of AI technology can induce changes in business operations, potentially influencing the demand for various categories of labor, including individuals with lower skill levels. Moreover, the finding also indicates the significance of mitigating any disparities in skills within the workforce. The potential impact of AI adoption on employment requirements may need reskilling and upskilling programs for those with lower skill levels to align their abilities with the changing demands of the labor market [38,39].

The causal relationship between inflation and low-skilled employment is uni-directional. The finding indicates that the inflation rate (INF) can be used to predict fluctuations in low-skilled employment. Changes in inflation rates may impact the demand for low-skilled labor. Inflation can impact labor costs in the long run. High inflation can result in increased prices for goods and services and higher wages. Inflation may impact businesses' decisions on hiring and employing low-skilled workers by potentially increasing their labor costs. Inflation impacts both the demand and supply sides of the labor market. Businesses may decrease hiring in response to inflation-induced increases in labor costs. Workers may alter their labor force participation in response to fluctuations in real wages (Anakpo and Kollamparambil, 2021; [40]).

Furthermore, foreign direct investment (FDI) has a causal relationship with low-skilled employment. Thus, previous fluctuations in FDI can serve as a reliable indicator for predicting future changes in low-skilled employment. Similarly, low-skilled employment has a Granger-causal relationship with foreign direct investment, suggesting that changes in low-skilled employment can be used to predict future changes in FDI. The bidirectional Granger causality suggests a mutual influence relationship between the two variables. Additionally, the finding that FDI has a causal relationship with low-skilled employment, and vice versa, implies that FDI and low-skilled employment are mutually dependent. Alterations in one variable can result in corresponding modifications in the other, thereby establishing a feedback loop. This reciprocal influence can profoundly impact economic dynamics. The implication is that higher levels of foreign direct investment (FDI) can stimulate job growth and generate income, thereby increasing domestic consumption and creating more opportunities for low-skilled workers (Coniglio et al., 2015; [23]).

### 5.7 Impulse response function

**Fig. 1:**
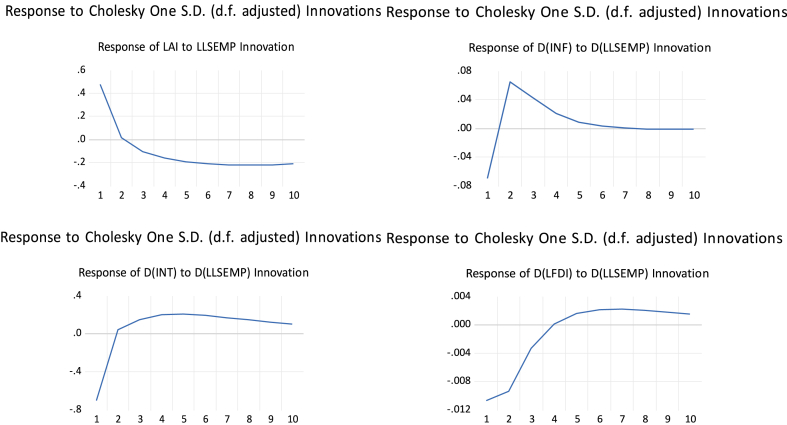
General Impulse Response Function (GIRF) results.

Source: Calculation by the authors

The impulse response functions trace the time paths of the effect of structural shocks on low-skilled employment (LoSEMP) in response to a unit shock to its explanatory variables. Fig. 1 shows the impulse response function results.

The response of artificial intelligence (AI) to low-skilled employment (LSEMP) demonstrates a positive shock from time 0 to 1 and a negative shock from time 2 to 10. The positive shock could mean a sudden rise in the influence or prevalence of artificial intelligence from time 0 to time 1. This scenario depicts a situation in which the adoption of AI technologies occurs rapidly, resulting in increased automation, improved efficiency, and the emergence of new job prospects in specific domains. The negative shock could mean a decline in the influence of artificial intelligence (AI) from time 2 to 10. This scenario reflects a decline in the initial optimism surrounding the impact of AI, potentially attributed to unanticipated challenges and job displacements. Consequently, certain low-skilled jobs that were previously established may be phased out [41].

Inflation (INF) response to low-skilled employment (LSEMP) demonstrates a negative shock from time 0 to 2, a positive shock from time 2 to 7, then narrowly sways to the negative region. The negative shock from 0 to 2 may be attributed to reduced consumer spending and economic uncertainty. Consequently, employers may reduce their hiring practices, affecting employment opportunities for individuals with lower skill levels. A decrease in demand for goods and services can result in businesses reducing production and implementing workforce reductions, which may include individuals with lower skill levels [32]. During the period of positive shock (2–7), there is an improvement in economic conditions. The positive shock may arise from increased consumer demand, increased economic activity, and creating more job opportunities for individuals with lower skill levels. Businesses expanding production to meet increased demand can lead to an increase in hiring, which positively affects low-skilled employment. Following an initial positive shock, there is a subsequent decline in the positive influence of economic conditions on inflation. The fall could result in reduced job growth and stagnant wages, especially for workers with lower skill levels (Anakpo and Kollamparambil, 2021).

Interest rate (INT) response to low-skilled employment (LoSEMP) demonstrates a negative shock from time 0 to 2, and a positive shock from time 3 to 10. The initial negative shock (0–2) may result in reduced borrowing costs for businesses and consumers. Reduced interest rates may stimulate borrowing, promoting higher expenditure and investment levels. Due to business investments and expansions, increased job opportunities may arise in low-skilled employment. During the period of positive shock (3–10), interest rates rise as a result of a more restrictive monetary policy. Higher interest rates may lead to reduced borrowing, potentially resulting in decreased business investment and consumer spending. The increasing caution of businesses regarding borrowing may have implications for their expansion strategies and consequently impact employment opportunities for individuals with low skills [34].

Foreign direct investment (FDI) response to low-skilled employment (LSEMP) demonstrates a negative shock from time 0 to 5, and a positive shock from time 5 to 10. The negative shock (0–5) could decrease international investment activity within the country. This decrease could affect job creation across different skill levels, including low-skilled employment. According to Bozsik et al. [36] reduced investment and delayed expansion plans by businesses, especially multinational corporations, may impact opportunities for low-skilled workers. During the period of positive shock, an increase in foreign direct investment may result in more significant inflows of international capital into the country. This rise could positively affect the economy, potentially increasing employment opportunities in various industries. Foreign investment in certain sectors can lead to increased demand for low-skilled labor, thereby benefiting workers with limited skills (Coniglio et al., 2015).

### 5.8 Variance decomposition

The variance decomposition analysis examines the contributions of different variables to the variation in low-skilled employment during the specified ten-year period. The data given emphasizes the impact of artificial intelligence, inflation, interest rates, and foreign direct investment on the fluctuations in low-skilled employment. Variance decomposition results are presented in Table 8.

**Table 8:** Variance decomposition.

**Variance Decomposition of LLoSEMP**						
**Period**	S.E.	LLSEMP	LAI	INF	INT	LFDI
**1**	0.005942	100.0000	0.000000	0.000000	0.000000	0.000000
**2**	0.009562	84.41601	4.354173	10.60764	0.386502	0.235672
**3**	0.012057	77.17227	5.107413	17.15702	0.247508	0.315790
**4**	0.013986	72.26818	5.096931	21.84454	0.458698	0.331653
**5**	0.015593	68.47690	4.925703	25.28312	0.997912	0.316364
**6**	0.016981	65.46928	4.750018	27.76289	1.730152	0.287656
**7**	0.018200	63.09486	4.613006	29.49634	2.539445	0.256348
**8**	0.019279	61.24390	4.523530	30.65662	3.347116	0.228843
**9**	0.020236	59.82217	4.479162	31.38394	4.106199	0.208534
**10**	0.021085	58.74769	4.473849	31.78941	4.792240	0.196812

The proportion of low-skilled employment influenced by artificial intelligence has steadily risen. In the second year, AI accounts for 4.354 % of the variance, indicating that this percentage of the decrease in variability of low-skilled employment can be attributed to AI. In year three, AI accounts for 5.107 % of the variance reduction, while in year ten, it accounts for 4.473 % of the variance reduction.

The proportion of variance in low-skilled employment attributed to inflation also rises as time progresses. In the second year, inflation accounts for 10.607 % of the reduction in variance, which gradually increases to 31.789 % by the tenth year. This proportion implies that fluctuations in inflation increasingly affect the decline in employment opportunities for individuals with limited skills.

Interest rates gradually increase the contribution to reducing variance in low-skilled employment. Interest rates explain 0.386 % of the variance reduction in year two, and this proportion slowly rises to 4.792 % by year ten.

The contribution of foreign direct investment (FDI) to the variation in low-skilled employment diminishes over time. FDI accounts for 0.235 % of the variance reduction in year two, decreasing to 0.196 % in year ten. This proposition suggests that the influence of foreign direct investment (FDI) on the decrease in fluctuations in low-skilled employment diminishes over time.

### 5.9 Diagnostic result

The results of the residuals diagnostic in Table 9 demonstrate the p-values of heteroscedasticity, normality, and autocorrelation test are above the five percent significance level. Therefore, we fail to reject the null hypothesis. In conclusion, residuals are homoscedastic, normally distributed, and have no serial correlation. Overall, the regression model meets the criteria of satisfaction.

**Table 9:** Diagnostic test.

**Tests**	**Type of Test**	**F-Statistic**	**p-value**	**Decision**
**Serial Correlation**	Breusch-Godfrey	1.7629	0.1883	Fail to reject $H_{0}$
**Heteroscedasticity**	Breusch-Pagan Godfrey	2.0798	0.1066	Fail to reject $H_{0}$
**Normality**	Jarque-Bera	0.3425	0.842592	Fail to reject $H_{0}$

Note: Significance levels are 1 %, 5 %, and 10 %, denoted by ∗∗∗, ∗∗, and ∗, respectively.

## 6 Conclusion and recommendation

This paper examined the relationship between artificial intelligence and low-skilled employment in South Africa and also considering various economic variables. The study utilized a VECM (Vector Error Correction Model) approach to analyze the dynamics among the variables: low-skilled employment, artificial intelligence investment, inflation, interest rate, and foreign direct investment.

The unit root test results indicate that all variables are integrated into the first difference, suggesting a need to consider their changes over time. Given this, the VECM method is deemed appropriate for the analysis. Lag criteria results suggest a logarithmic transformation for the variables. The Johansen cointegration test supports the existence of cointegration among the variables, indicating a long-term equilibrium relationship among them. This result implies that there are stable relationships driving the interplay between these factors over time.

In the short run, the study finds that artificial intelligence investment and inflation negatively and significantly impact low-skilled employment. In contrast, interest rates and foreign direct investment exhibit positive and significant effects. In the long run, the negative and significant impact of artificial intelligence investment and inflation on low-skilled employment is reinforced. Interest rate, however, is found to be insignificant in influencing low-skilled employment, while foreign direct investment continues to have a positive and significant effect.

Granger causality tests reveal the directional relationships between the variables. Artificial intelligence investment and inflation are shown to Granger cause low-skilled employment, indicating a unidirectional influence. Foreign direct investment Granger causes low-skilled employment; interestingly, a bidirectional relationship exists between foreign direct investment and low-skilled employment.

Impulse response analysis indicates mixed responses among all the variables, highlighting the complex dynamics at play. Variance decomposition underscores the considerable influence of artificial intelligence investment, inflation, and interest rate on explaining the variance in low-skilled employment. The diagnostic tests for serial correlation, heteroscedasticity, and normality suggest that the model's assumptions are met, enhancing the reliability of the findings.

The study's findings have important implications for policymakers and stakeholders aiming to navigate the challenges and opportunities presented by the evolving economic landscape driven by technological advancements. Given the adverse effects of artificial intelligence investment on employment opportunities for individuals with low skills, it is imperative to implement proactive strategies to enhance this workforce's skills and knowledge. Policymakers should prioritize implementing training programs to equip workers with the necessary skills to adapt to the evolving job market influenced by technological advancements. Policymakers should aim to find a middle ground by embracing technological advancements and addressing potential adverse impacts on low-skilled employment. A solution may entail monitoring the implementation of automation technologies and establishing policies to mitigate labor market disruptions. Given the substantial effect of artificial intelligence investment on low-skilled employment, policymakers must prioritize investments in research and innovation, such as endorsing research and development endeavors that prioritize the creation of economically beneficial technologies with favorable employment implications.

## CRediT authorship contribution statement

**Fiyinfoluwa Giwa:** Writing – review & editing, Writing – original draft, Visualization, Software, Resources, Project administration, Methodology, Investigation, Formal analysis, Data curation, Conceptualization. **Nicholas Ngepah:** Validation, Supervision, Funding acquisition, Conceptualization.

## Data availability statement

The data and content used in this study were obtained from publicly accessible online platforms and repositories. No permissions were necessary for their utilization, as they fall under the public domain and are subject to open-access policies.

## Declaration of Competing interest

The authors assert that there are no conflicts of interest that could impact the objectivity of this research. The present study was carried out with a strong commitment to upholding integrity and transparency, ensuring that the findings' validity remained unaffected by any potential financial or personal relationships.
